# Genomic epidemiology of *Mycobacterium abscessus* in a Canadian cystic fibrosis centre

**DOI:** 10.1038/s41598-022-19666-8

**Published:** 2022-09-27

**Authors:** Nicholas Waglechner, Elizabeth Tullis, Anne L. Stephenson, Valerie Waters, Fiona McIntosh, Jennifer Ma, Frances B. Jamieson, Marcel A. Behr, Jane Batt, Robyn S. Lee

**Affiliations:** 1 Dalla Lana School of Public Health, University of Toronto, 155 College St., Toronto, ON M5T 3M7 Canada; 2 Adult Cystic Fibrosis Program, Division of Respirology, St. Michael’s Hospital, Unity Health Toronto, Toronto, ON Canada; 3 Li Ka Shing Knowledge Institute, St. Michael’s Hospital, Toronto, ON Canada; 4 Institute of Health Policy, Management and Evaluation, University of Toronto, Toronto, ON Canada; 5 Department of Pediatrics, Division of Infectious Diseases, Hospital for Sick Children, 555 University Avenue, Toronto, ON M5G 1X8 Canada; 6 Infectious Diseases and Immunity in Global Health Program, Research Institute of the McGill University Health Centre, Montreal, QC Canada; 7 McGill International TB Centre, McGill University, Montreal, QC Canada; 8 Public Health Ontario, Public Health Ontario Laboratories, 661 University Avenue, Suite 1701, Toronto, ON M5G 1V2 Canada; 9 Department of Epidemiology, Biostatistics and Occupational Health, McGill University, Montreal, QC Canada; 10 Keenan Research Center for Biomedical Science, St. Michael’s Hospital, Toronto, ON Canada; 11 Tuberculosis Program, St. Michael’s Hospital Unity Health Toronto, Toronto, ON M5B 1WB Canada; 12 Center for Communicable Disease Dynamics, Harvard School of Public Health, Boston, MA USA

**Keywords:** Bacterial infection, Cystic fibrosis, Epidemiology, Genome informatics

## Abstract

The *Mycobacterium abscessus* complex causes significant morbidity and mortality among patients with Cystic Fibrosis (CF). It has been hypothesized that these organisms are transmitted from patient to patient based on genomics. However, few studies incorporate epidemiologic data to confirm this hypothesis. We longitudinally sampled 27 CF and 7 non-CF patients attending a metropolitan hospital in Ontario, Canada from 2013 to 2018. Whole genome sequencing along with epidemiological data was used to evaluate the likelihood of transmission. Overall, the genetic diversity of *M. abscessus* was large, with a median pairwise distance (IQR) of 1,279 (143–134) SNVs between all Ontario *M. abscessus* isolates and 2,908 (21–3,204) single nucleotide variants (SNVs) between *M. massiliense* isolates. This reflects the global diversity of this pathogen, with Ontario isolates widely dispersed throughout global phylogenetic trees of each subspecies. Using a maximum distance of 25 SNVs as a threshold to identify possible transmission, we identified 23 (of 276 total) pairs of closely-related isolates. However, transmission was probable for only one pair based on both genomic and epidemiological data. This suggests that person-to-person transmission of *M. abscessus* among CF patients is indeed rare and reinforces the critical importance of epidemiological data for inferences of transmission.

## Introduction

Some of the most prevalent organisms identified in CF and non-CF patients with Nontuberculous Mycobacteria (NTM) pulmonary disease belong to the *Mycobacterium abscessus* complex^1,2^. *M. abscessus* infections require extended periods of antimicrobial therapy, contribute to lung function decline and premature mortality, and if poorly controlled are a contraindication for lung transplantation^3,4^. This complex is comprised of three subspecies, *M. abscessus* subsp. *abscessus*, *M. abscessus* subsp. *massiliense*, and *M. abscessus* subsp. *bolletii*^5^, or simply *M. abscessus*, *M. massiliense*, *M. bolletii*.

The risk of acquiring an *M. abscessus* complex infection from the environment and potential role of person-to-person transmission are poorly understood. *M. abscessus* has been found in water from household and hospital plumbing, and soil^6–9^ but few direct links have been established between environmental sources and infection^10^. Acquisition by transmission between patients has also been proposed^11,12^. Evidence for this is conflicting; multiple studies have reported clusters of highly similar genomes from different CF centres, attributing this similarity to person-to-person transmission^12–15^, however, epidemiological contact data was largely lacking. Genomic studies including epidemiological data have refuted patient-to-patient transmission^16–21^. To address this limitation and further evaluate potential transmission between CF patients, we conducted a retrospective study of *M. abscessus* in a large Canadian CF centre where detailed hospitalization and clinic attendance data were available.

## Materials and methods

### Sample collection, clinical and demographic data

Public Health Ontario Laboratory, the reference laboratory for *M. abscessus* in Ontario, provided frozen isolates obtained from sputum specimens of 27 adult patients with CF receiving routine clinical care at St. Michael’s Hospital, Toronto, from 2013 to 2018, representing all *M. abscessus* isolates from this centre during the study period. We included a convenience sample of isolates from seven non-CF patients obtained between 2013 and 2018. Each patient was assigned a number (CF patients 1–30, non-CF patients 31–37) while each isolate was assigned an ascending identifier (i.e. A to Z, 2A to 2Z, etc.) in order of collection. Isolates were grown using standard methods^22^. Isolates were resurrected in culture on Middlebrook 7H10 agar and plate sweeps were collected for DNA extraction using the van Soolingen method^23^. Genomic DNA was quantified using the Quant-iT PicoGreen dsDNA Assay (ThermoFisher Scientific, Massachusetts, USA). Library preparation and sequencing was performed at the McGill University/Génome Québec Centre d'expertise et de services Sequencing Centre (Montreal, QC) in 2 × 250 bp paired-end format on the Illumina MiSeq platform.

Patient demographic and clinical data and dates of clinic visits, hospitalizations, emergency room (ER) visits, and Pulmonary Function Testing (PFT) were obtained from the Canadian Cystic Fibrosis Registry for CF patients or the St. Michael’s Hospital electronic data warehouse Soarian for non-CF patients.

### Sequencing and quality control

Isolates were sequenced in 2 × 250 bp format on the Illumina MiSeq platform. Snakemake (v6.0.3)^24^ was used to develop and run the analysis workflow, available at https://github.com/waglecn/mabs. Raw sequencing reads were processed with Trimmomatic (v0.39)^25^ for sequencing adapter removal, quality trimming and filtering, and for minimum length (options “ILLUMINACLIP:2:30:10 SLIDINGWINDOW:4:15 MINLEN:36”). Kraken2 contamination results were computed using a prebuilt minikraken database (available from ftp://ftp.ccb.jhu.edu/pub/data/kraken2_dbs/old/minikraken2_v2_8GB_201904.tgz) containing Refseq bacteria, archaeal, viral and human sequences. At least 75% of the reads for each isolate were required to have a primary taxonomic assignment to *M. abscessus* at the species rank, with no more than 1% of reads assigned to any other species to be considered uncontaminated^26^. Each sample was assembled de novo using Shovill (default parameters, v1.1.0, https://github.com/tseemann/shovill). We identified assembly contamination or mixed isolates if the assembled genome length was larger than 5,700,000 bp. Reads passing QC were aligned to the *M. abscessus* ATCC 19977 reference genome using bwa-mem (default parameters, v0.7.17-r1188)^27^. We required a mean depth ≥ 40 × to include a sample in single nucleotide variant (SNV) analysis (Supplementary Table 1).

### Erm(41) detection

We examined the *erm*(41) allele present in the genome assembly of each isolate with the translated Basic Local Alignment Search Tool (tblastn) using the reference *erm*(41) protein sequence (accession ABW06859.1)^28^. A full-length allele (138 amino acids) can putatively indicate *M. bolletii* or *M. abscessus*, while presence of a truncated allele (30 amino acids) indicate *M. massiliense*, however presence of the truncated allele is not definitive for subspecies identication of *M. bolletii* or *M. massilisense*^29,30^.

### Subspecies identification

A *k*-mer based phylogeny of assembled genomes, including the reference genomes for *M. abscessus* ATCC 19977, *M. massiliense* CCUG 48898, and *M. bolletii* BD (accessions GCF_000069185.1, GCF_000497265.2 and GCF_003609715.1, respectively) was produced using mashtree^31^ and rooted using *Mycobacterium tuberculosis* H37Rv (GCF_000195955.2) as outgroup. The closest reference sharing a most recent common ancestor (MRCA) in this tree was used to assign a putative subspecies to each isolate. We used the presence or absence of a full-length *erm*(41) allele to further identify *M. bolletii* isolates*.*

### MLST assignment

The mlst software (v2.19.0) was used to compare each assembled sample genome against the bundled seven locus (*argH*, *cya*, *gnd*, *murC*, *pta*, *purH*, and *rpoB*) PubMLST^32^ schemes ‘mabscessus’, for *M. abscessus* isolates and ‘mmassiliense’ for *M. massiliense*, as determined by the closest MRCA in the mashtree^31^ phylogeny, and assigned a sequence type to each isolate (available at https://github.com/tseemann/mlst).

### Variant calling, filtering

The plasmid sequence in the *M. abscessus* ATCC 19977 reference was excluded so only the chromosomal sequences for each reference were used for variant detection and phylogenetics. Reads for each isolate were mapped to its assigned subspecies reference using bwa-mem with default parameters^27^, followed by local realignment around indels. The RealignerTargetCreator and IndelRealigner (Genome Analysis ToolKit v3.8) was used to identify insertion-deletion sites as targets for read realignment, using default parameters^33^. Alignments were filtered using samtools (v1.9)^34^. Only primary alignments of reads having fewer than 20% soft-clipped bases and a mapping quality (MAQ) above 30 were retained.

Variants were called using bcftools (v1.9) using bases with a base alignment quality (BAQ) above 30, minimum depth (DP) of 10, a minimum alternate allelic depth (AD) of 1, a Phred-scaled quality of 50 and strand bias p-value (SP) of 45^35^. Following this, regions of potential recombination were identified using the Gubbins software (v2.4.1) requiring a minimum number of 20 SNVs in the default sliding window and up to 20 iterations^36^. Recombinant or zero-coverage regions in any isolate, as well as regions coding for sequences annotated as poly-Glu or poly-Pro/Glu (PE and PPE) proteins, were masked using bedtools^37^ to generate an alignment of core-SNVs for each subspecies. Recombinant regions, regions of zero coverage, and PE-PPE regions were masked.

### Maximum-likelihood phylogeny

A maximum-likelihood phylogeny and pairwise distance matrix for each subspecies was produced from each core SNV alignment. The pairwise SNV distance was produced using snp-dists (https://github.com/tseemann/snp-dists). Trees were constructed with IQ-tree (v2.0.3) using automatic model selection and 10,000 ultra-fast bootstraps^38^. Up to 2,000 iterations of the ultra-fast bootstrap procedure were used, adding increments of 200 additional iterations as necessary, until convergence was achieved. Clades were labeled based on grouping of patient isolates with a minimum bootstrap support of 80%.

### Transmission criteria

We used the upper 95th percentile of pairwise SNV distances from within-patient isolates to set a genomic distance threshold to consider transmission. Pairs of isolates from different patients that had genomic distances above this threshold were not evaluated further. Pairs of isolates from different patients that were within this threshold were considered ‘possible’ transmission pairs and underwent detailed epidemiological and clinical review.

Transmission was considered to be ‘probable’ if patient contact was recorded, i.e. had attended the same clinic or had PFT on the same day, within six months preceding the second patient’s first positive *M. abscessus* culture. While hospital admission data was reviewed, it was considered unlikely that transmission would have occurred between two patients hospitalized with *M. abscessus.* At this facility, all admitted CF patients are in single-patient rooms or shared rooms exclusively with non-CF patients, who are not diagnosed with any infectious disease and/or bronchiectasis. Additionally, per Canadian CF guidelines, they are placed on contact precautions; all hospital staff are required to don and doff personal protective equipment (gowns and gloves), wash hands before entry and on exiting rooms, and any shared equipment is cleaned between use (including spirometry, done in patient rooms), and rooms undergo a terminal cleaning at discharge. Finally, when leaving their rooms for any other testing (e.g., chest imaging), patients are also required to wear a surgical mask.

Transmission was considered ‘unlikely’ if contact occurred more than six months prior to the second patient’s first positive *M. abscessus* culture, and/or if intervening negative *M. abscessus* microbiology results were obtained before the second patient’s first positive isolate.

### Global sequences

Data from 6,260 global *M. abscessus* isolates were obtained from the National Center for Biotechnology Information (NCBI) sequence read archive (SRA).

### Ethics

All patient data and bacterial samples were collected as part of routine clinical care and analyzed in a non-nominal fashion, therefore the requirement for informed consent was waived when this study was approved by the University of Toronto Office of Research Ethics (HPR-00018161) and St. Michael’s Hospital Research Ethics Office (REB#15-399). All work was performed in accordance with the relevant guidelines and regulations.

## Results

Thirty patients with CF were diagnosed with *M. abscessus* complex infections between 2013 and 2018 at the study centre, however, no isolates obtained from three patients (2,13, and 26); these are not included in any totals. (Table 1). A total of 228 isolates were obtained from sputum samples collected from the remaining 27 patients (Table 2). 56 CF isolates were not available for sequencing as they failed to be resurrected in culture from the frozen stocks or were found to be contaminated. This includes all isolates available from patient 6 (five isolates), 21 (one isolate), and 24 (three isolates). The remaining 172 CF isolates were sent for sequencing, however three isolates were found to be too contaminated for nucleic acid extraction or library preparation, while the remaining 169 were each sequenced at least once. Of the CF isolates that were sequenced, 118/169 isolates (69.8%) were sequenced once, while 51 isolates (30.1%) required repeated attempts at sequencing due to contamination or insufficient coverage. 19 of these 51 (37.2%) were regrown (these isolates bear the suffix ‘_2’ in their identifiers) while 32/51 (62.8%) were sequenced twice after being resurrected. After sequencing, 152/169 (89.9%) isolates passed QC, representing 24 of the 30 CF patients. Seven non-CF patients were included for context, who produced 10 positive isolates during this time (Table 2). Of these, two isolates were not available for sequencing (including the only isolate from patient 33), while one isolate was found to be too contaminated for sequencing. The remaining 7/10 isolates, representing 6 non-CF patients, were available, sequenced, and passed QC.

**Table 1:** Description of patients.

All patients (n = 33)	
Median age, years (IQR)	31 (22–45)
Sex, M/F	21/12
**CF (n = 27)**	
Median (IQR) age, years	26 (21–38)
Median (IQR) age at CF diagnosis, years (IQR)	1 (0–10)
Sex, M/F	17/10
No. (%) treated for NTM	8 (30)
No. (%) diagnosed with *CFRD	5 (19)
No. (%) with pancreatic insufficiency	21 (78)
Median (IQR) BMI at first *M. abcessus* positive	22.1 (19.6–24.9)
**Non-CF (n = 7)**	
Median (IQR) age, years	60 (49–66)
Sex, M/F	5/2
No. (%) treated for NTM	0 (0)

*CFRD: cystic fibrosis-related diabetes.

**Table 2:** Isolates included and corresponding patient numbers.

All isolates (n = 238)	Isolates
CF isolates (%)	228 (95.8)
Non-CF isolates (%)	10 (4.2)
**CF isolates (n = 228 from 27 patients)**	
Grown (%)	172 (75.4)
Failed to grow (%)	56 (24.6)
**Grown (n = 172 from 24 patients)**	
Contaminated before sequencing (%)	3 (1.7)
Sequenced (%)	169 (98.3)
**Sequenced (n = 169 from 24 patients)**	
Isolates passing QC (%)	152 (89.9)
Isolates failing QC (%)	17 (10.1)
**Final patients included/excluded based on growth and sequencing QC**	
Number of patients with at least one isolate included	24
Number of patients excluded due to isolates failing QC	3
**Non-CF isolates (n = 10 from 7 patients)**	
Grown (%)	8 (80)
Failed to grow (%)	2 (20)
**Grown (n = 8 from 6 patients)**	
Contaminated before sequencing (%)	1 (10)
Sequenced (%)	7 (87.5)
**Sequenced (n = 7 from 6 patients)**	
Isolates passing QC (%)	7 (100)
Isolates failing QC (%)	0 (0)
**Final patients included/excluded based on growth and sequencing QC**	
Number of patients with at least one isolate included	6
Number of patients excluded due to isolates failing QC	0

The majority of isolates that failed QC were rejected for insufficient coverage (12/17, 70.5%) and one (20-E) failed due to contamination (1/17, 5.8%) (Supplementary Table 3). Four out of 17 QC-failing isolates (17-E, 17-F_2, 29-2L, and 29-3L, 23.5%) appeared to have a larger assembled genome size than expected compared to the other isolates and the reference genomes used in the analysis. Kraken 2 results indicated that these isolates did not have contaminating sequence at a taxonomic level higher than species and suggests these patients were potentially infected with a mixed population of *M. abscessus*^3^. The six patients with CF (3 males, 3 female) that were not included in the analysis (due to not having isolates successfully cultured and/or sequenced but failing QC) were similar in age to those who were included (median age at first *M. abscessus* positive infection of 22.0 years vs. 25.9 years for excluded and included patients, respectively; Wilcoxon rank sum test *p* = 0.23).

### Subspecies identification

**Figure 1:**
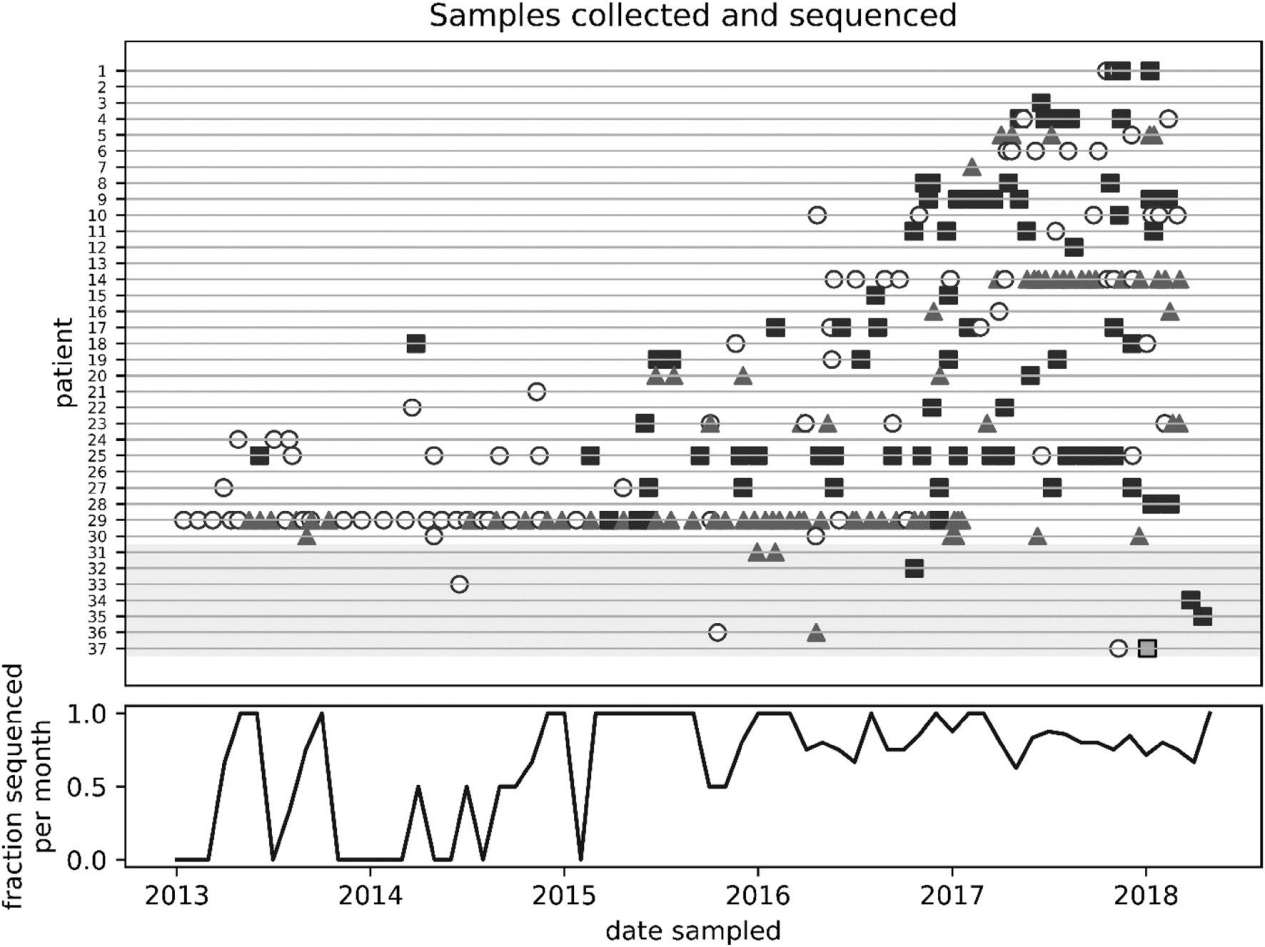
Sampling date and subspecies assignment. Isolates collected from 2013 to 2018 from 30 patients with CF and 7 non-CF patients. (**a**) Isolates either could not be regrown or used for sequencing (open circles) or were sequenced and successfully assigned a subspecies (*M. abscessus*-squares, *M. massiliense*-triangles, *M. bolletii*-filled grey square). More isolates were available from patients with CF (1–30) than non-CF patients (31–37, grey background). (**b**) Fraction of available isolates sequenced by month.

Most isolates were identified as either *M. abscessus* or *M. massiliense* (Supplementary Table 1). Among patients with CF, 74 isolates were identified as *M. abscessus* and 78 isolates were *M. massiliense*. In non-CF patients, three isolates each were *M. abscessus* and *M. massiliense.* A single isolate was classified as *M. bolletii* (37-B) from a non-CF patient. Nearly all isolates from an individual belonged to a single subspecies (Fig. 1). Two patients with CF (23 and 29) were infected by both *M. abscessus* and *M. massiliense* at different times. The first isolate from patient 23, from 2015, was *M. abscessus*, while the subsequent eight isolates from 2015 to 2018 were *M. massiliense*. Of 43 isolates from patient 29 (from 2013 to 2017), 41 were *M. massiliense* except for two *M. abscessus* isolates (29-2I, 29-2 K) obtained in April and May 2015.

### Utility of MLST assignment

**Figure 2:**
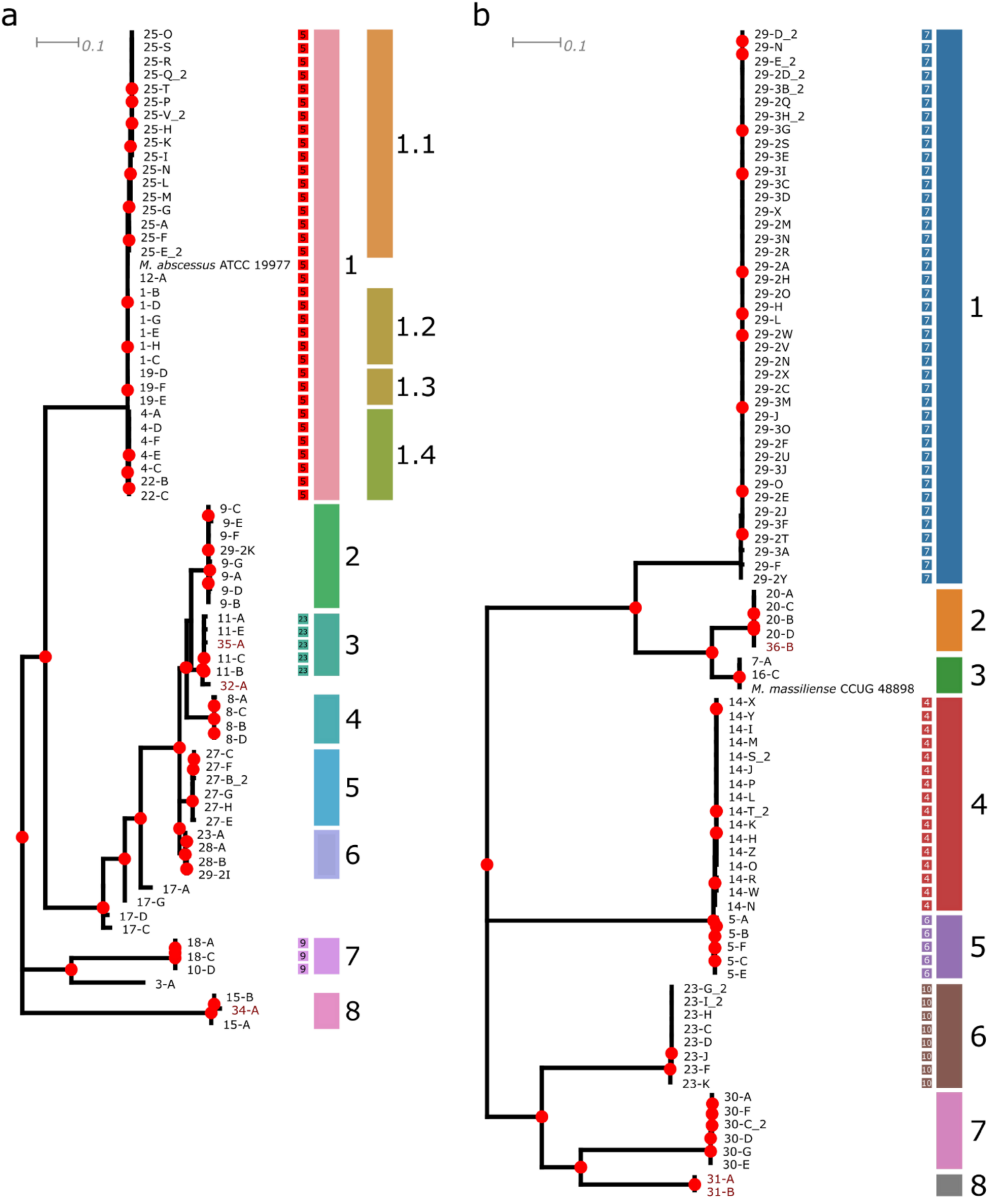
Subspecies maximum likelihood phylogeny. These phylogenies were constructed from the recombination-masked SNV alignment, and drawn using the midpoint root. Bootstrap support > 80% is indicated on nodes with red circles. The seven locus (*argH*, *cya*, *gnd*, *murC*, *pta*, *purH*, and *rpoB*) PubMLST sequence types (ST) are indicated in coloured squares where an existing ST could be assigned (see Methods). Isolates from patients with CF are indicated in black, while those from non-CF patients are indicated in dark red. **a** *M. abscessus. ****b**** M. massiliense*.

Multi-locus sequence typing broadly corresponds with the clades we have indicated in both trees (Main text Fig. 2a,b), but provides limited resolution to discriminate between isolates from different patients (for example, in *M. abscessus* clade 1) compared to whole genome sequencing data^13^. Across the two trees, no sequence type could be assigned to 45 isolates from 16 different individuals.

### Within-host diversity of *M. abscessus*

84,855 SNVs were identified from 74 M*. abscessus* isolates and 125,064 SNVs from 78 M*. massiliense* isolates. After adjusting for recombination 3,452/84,855 (4.06%, *M. abscessus*) and 6,603/125,064 (5.26%, *M. massiliense*) SNVs were used for phylogenetic and pairwise distance analysis (Fig. 2a,b, Supplementary Table 2–3, Supplementary Figs. 2–3). The median pairwise distance (IQR) to the reference for *M. abscessus* was 1,066 (12–1,322) SNVs compared to 1,668 (1,662–3,076) SNVs for *M. massiliense*. Since only a single *M. bolletii* isolate was available in the Ontario data a tree could not be estimated for this subspecies. The median pairwise distance [IQR] of *M. abscessus* isolates was 1,279 [143–1,340] SNVs, and for *M. massiliense* 2,908 [21–3,204] SNVs.

The best model for both alignments chosen using the Bayesian information criterion (BIC) was the transversion model (TVM) where substitution frequency of GT = CT, empirical base frequencies (+ F) measured from the alignment, and the ascertainment bias correction (+ ASC). Both trees used the FreeRate model of among-site rate heterogeneity. The *M. abscessus* data fit a 3-category model (+ R3) best while the *M. massiliense* alignment fit a 4-category model best (+ R4).

**Figure 3:**
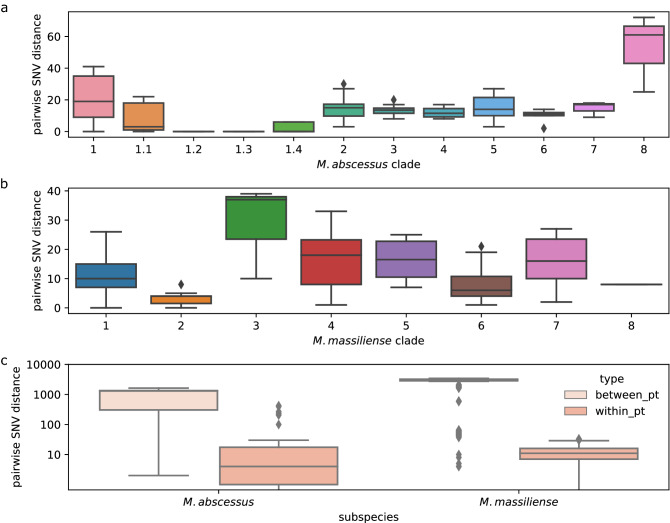
Summary of pairwise SNV distances. Recombination-masked SNV distances for the indicated clades were computed for each subspecies and clade in Fig. 2. Lines and boxes indicate the median and interquartile range (IQR), while whiskers extend to 1.5 × the IQR. Colours match the indicated clades in the  Fig. 2. Where missing, median lines coincide with upper quartiles. (**a**) *M. abscessus.* (**b**) *M. massiliense*. **c** Within- and Between-patient pairwise SNV distances by subspecies.

Isolates from the same patient clustered together with the exception of those from patient 17 and 29 in *M. abscessus* (Fig. 2a). The four isolates from patient 17 were collected over 22 months, had median pairwise distance (IQR) of 223 (207–336) SNVs, and did not form a cluster in the phylogeny. These isolates were also found to have partial MLST alleles (Supplementary Table 1). The *M. massiliense* isolates from patient 29 were all found to be ST 7, while the two *M. abscessus* isolates from that patient (29-2I and 29-2K) were both novel STs that differed at the *cya* allele (Supplementary Table 1). Clades were well-supported (bootstrap values 98–100). The pairwise distance within indicated clades ranged from 0 (clade 1.3, 1.4) to 68 SNVs (clade 8) in *M. abscessus* and from 4 (clade 2) to 38 SNVs (clade 3) in *M. massiliense* (Fig. 3a,b).

### Person-to-person transmission

The median pairwise distance [IQR] for between patient isolates for *M. abscessus* was 1303 [305–1,344] SNVs, and for *M. massiliense* was 3,056 [2,855–3,214] SNVs. Conversely, the median pairwise distance for within-patient isolates for *M. abscessus* was 4 [1–18] SNVs, and for *M. massiliense* was 11 [7–16] SNVs (Fig. 3). As 95% of within-patient isolate pairs were separated by fewer than 25 and 23 SNVs (*M. abscessus* and *M. massiliense,* respectively), we considered isolates separated by 25 or fewer SNVs to be closely related and possibly indicative of transmission, warranting further epidemiologic investigation.

Using genomics alone, we identified 23 pairs of isolates within the 25 SNV threshold, involving 21 M*. abscessus* and two *M. massiliense* patient pairs (rationale for these classifications is provided in Supplementary Table 4). Eleven of these 23 pairs had contact in hospital, however only two pairs had contact in the clinic in the six months before the second patient’s first positive result. Specifically, patient 4 had contact with both patient 1 and patient 22 (Supplementary Fig. 4). We ruled out transmission from patient 4 to patient 1 because patient 1 produced eight *M. abscessus*-negative sputum samples post-contact. Only patients 4 and 22 were epidemiologically linked with no intervening negative microbiology results, suggestive of probable transmission. The remaining nine patient pairs were considered unlikely to be transmission as contact occurred more than six months, in some cases years, before the second patient became positive, where there were one or more negative *M. abscessus* microbiology results post-contact, or when one or both patients were hospitalized (a scenario where encountering one another was extremely improbable).

### Multiple subspecies infection

Two patients had infections from two subspecies (patient 29 and 23). For patient 29, isolates 29-2I and 29-2K (*M. abscessus*) were collected within two months of each other while the remaining 41 isolates from patient 29 were all *M. massiliense*. Isolates 29-2I and 29-2K were separated by 267 SNVs and unlikely to have been acquired from the same source. For patient 23, isolate 23-A was *M. abscessus* while subsequent isolates were *M. massiliense* Isolate 23-A was closely related (2 to 12 SNVs apart) to isolates from patients 28 and 29, however, patient 23 had no documented contact in clinic with these patients.

### Comparison of CF versus non-CF isolates

Non-CF isolates did not form a group separate from CF isolates; instead, they were interspersed in the phylogenetic trees, suggesting specific clades of *M. abscessus* do not preferentially infect patients with CF. Isolate 35-A (non-CF) was most closely related to isolates from patient 11 (CF) (median pairwise distance 16 SNVs, no epidemiologic link between patients), while 32-A (non-CF) was the next most closely related to these isolates (median pairwise distance 72 SNVs [IQR 71–73]). Isolate 34-A (non-CF) was most closely related to the two isolates from patient 15 (CF) (61 and 72 SNVs, respectively). Among the *M. massiliense* isolates, 36-B (non-CF) was most closely related to isolates from patient 20 (CF) (median pairwise distance 5 SNVs [IQR 4–6], no epidemiological link), while the two isolates from patient 31 (non-CF) were most closely related to those from patient 30 (CF) (median pairwise 1,904 SNVs [IQR 1,900–1,906]).

### Global diversity of the *M. abscessus* complex

Sequencing data for 3,711 of 6,260 M*. abscessus* complex isolates (59.3%) obtained from the NCBI SRA passed QC. Combined with the Ontario isolates, we generated global core SNV phylogenies with a total of 2,669 M*. abscessus*, 983 M*. massiliense*, and 219 M*. bolletii* isolates, respectively (Supplementary Table 5–6). Adjusting for recombination proved to be computationally intractable for global *M. abscessus* and *M. massiliense.* Because of this, unadjusted core SNV phylogenies are presented for each subspecies (Supplementary Figs. 5–7), along with an adjusted *M. bolletii* phylogeny in Supplementary Fig. 8. According to the Bayesian Information Criterion (BIC), the best model for the recombination adjusted *M. bolletii* alignment was GTR + F + R2, while for the unadjusted alignment it was TVM + F + R2. For both the unadjusted global *M. abscessus* and *M. massiliense* alignments, BIC identified TVM + F + R6 as the best fitting model.

**Figure 4:**
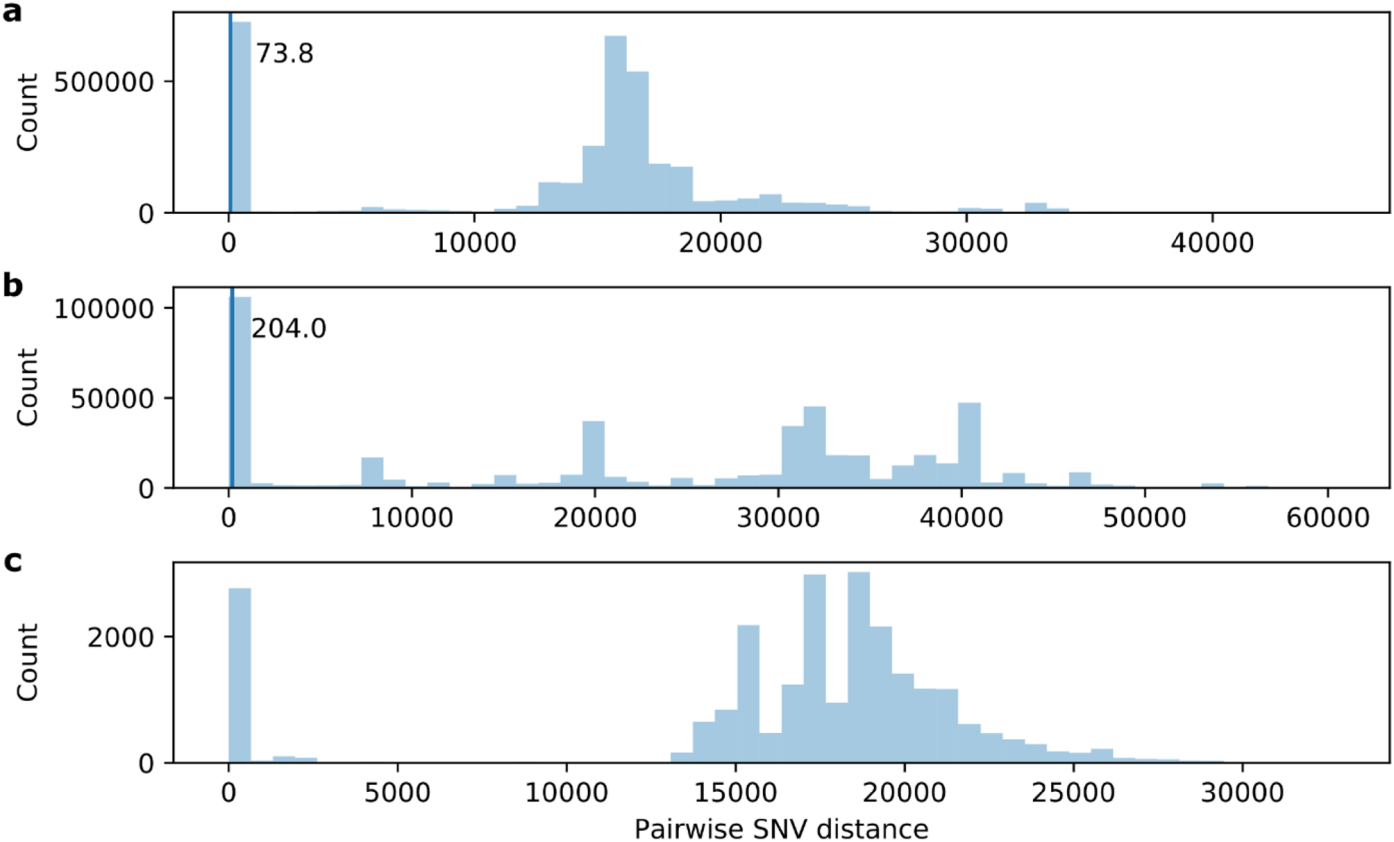
Distribution of unadjusted pairwise SNV distances of global *M. abscessus* isolates. The 95th percentile of pairwise within-patient SNVs of the Ontario isolates is indicated by vertical lines, but could not be calculated for *M. bolletii.* (**a**) *M. abscessus* (**b**) *M. massiliense* (**c**) *M. bolletii.*

The median (IQR) unadjusted within-patient pairwise distance for Ontario *M. abscessus* isolates in the global context was 4 (1–18) SNVs, similar to the values obtained for the recombination adjusted Ontario isolates alone. For Ontario *M. massiliense* isolates the unadjusted median (IQR) within-patient pairwise distance was 104 (57–161) SNVs, larger than when considering the Ontario isolates alone, but smaller than the Ontario isolates alone unadjusted for recombination (median (IQR) 235 (124–362) SNVs). The isolates from Ontario were interspersed throughout the global subspecies trees and did not cluster together. The distribution of pairwise SNV distances for these global sequences are shown in Fig. 4.

## Discussion

Among 276 pairs of adult patients with *M. abscessus* infection who attended this Ontario CF centre over a five-year period, we found only one probable transmission event (< 1% of all pairs). This suggests transmission in this patient population is rare, consistent with the work of others studying adult and pediatric CF patients where both genomic and epidemiological contact data were available^16–18,39^. This has important implications for infection control; while Canadian CF infection prevention guidelines do not currently recommend negative pressure rooms for patients with *M. abscessus* infection^40^, some centres (including ours) have implemented this measure in order to reduce the hypothetical risk of transmission. At our centre, as described in the Methods, multiple additional measures were also implemented to reduce risk of spread. The presence of different measures at different centres makes it difficult to compare reports of suspected patient to patient transmission of *M. abscessus*, and to assess the role of indirect transmission from HCWs^20^. While we cannot discount the possibility of transmission via health care workers, in light of the precautions in place at our facility and the data we present, we consider this risk to be low.

Studies on potential transmission of *M. abscessus* complex infections have provided conflicting evidence^12–14,16–18^. Dominant clones of *M. abscessus* have been seen in CF patients attending distant, even intercontinental, CF centres^12,13,41^. This global dissemination has been attributed to a pre-existing reservoir of *M. abscessus* in the lungs of smokers, and the colonization of a new reservoir in the lungs of CF patients experiencing major treatment improvements in the 1960s coinciding with the simultaneous emergence of the dominant circulating clones hypothesized to result in person-to-person transmission^42^. Complicating this, the supporting studies either lacked multi-year longitudinal within-patient sampling or robust epidemiological linkage, making it difficult to determine what, if any, role transmission played. In agreement with others^18^, we show WGS data is necessary but not sufficient to establish patient-to-patient *M. abscessus* transmission, as only one of the 23 patient pairs we considered to be possible transmission were actually in clinic the same day where transmission could have occurred. While it is possible that the remaining 22 patient pairs with < 25 SNVs between isolates had contact outside the hospital, the genomic diversity of *M. abscessus complex* across our patient population suggests that person-to-person transmission is not a driving factor for this organism.

Our work has many strengths. During the study period, all patients at our facility were routinely tested for *M. abscessus* infection at every visit, or at minimum annually, allowing us to capture all new infections. This reduces the probability of missed transmission events between patients, particularly as we have incorporated all available isolates from each patient, providing greater confidence in our findings. Inclusion of all longitudinal isolates allowed us to critically evaluate within-host diversity in our dataset and determine an appropriate SNV threshold for transmission. This analysis builds on previous reports of *M. abscessus* in patients with CF where 90% and 99% of isolates obtained from the same patient had fewer than 20 and 38 SNVs, respectively^12,13^. Another strength lies in the analytic approach; rather than using a single reference for a combined analysis, as in most previous genomic epidemiology studies of this complex, we conducted analyses of each subspecies separately using a complete reference genome for each. This improved our transmission analyses as we increased the proportion of sites present in our isolates and their respective reference genomes available for analyses. Finally, in addition to genomic data, we had access to robust epidemiologic data collected over the same study period, critical to rule in transmission.

Our study has several limitations. We were not able to sequence ~ 26% of the positive isolates from the patients with CF, primarily due to loss or failure to regrow isolates. However, we were able to include isolates from 24 of 30 patients with CF (80%) over the course of the study period. While we may have missed transmission involving the remaining six patients, this seems unlikely given the genetic diversity present in our dataset. Our results provide evidence that the dominant organism recovered from sputum can change at least at the subspecies level, and has been described by others^19,20^. Growing evidence suggests that single bacterial colonies isolated from sputum samples do not reflect the total diversity of *M. abscessus* complex subclones colonizing individuals with CF^43^. While we included multiple longitudinal isolates from each patient, where available, genomic DNA was extracted from plate sweeps and not individual clones^44^. This could potentially result in a mixture of strains being sequenced from a single specimen, as may be the case observed with patient 17’s isolates. Detecting a mixture of related subclones requires sequencing at higher depth than available for our isolates.

Another limitation is a lack of complete antibiotic treatment history for patients, which may help explain our observations that the dominant subspecies isolated from two patients changed over time. As *M. abscessus* and *M. massiliense* are partially differentiated by the presence or absence of an intact *erm*(41) allele, macrolide treatment may provide temporary selection for resistant *M. abscessus*. In addition, little is known about the time from exposure to first positive culture for *M. abscessus*; we assumed that that a positive culture would be obtained within six months of contact, however, this may underestimate potential transmission^9^. We also lack data on patient interactions outside the clinic, however as only 21 (7.6%) *M. abscessus* and 2 (0.7%) *M. massiliense* (out of 276 possible) patient pairs had isolates with genetic distances close enough to consider transmission possible and CF patients are encouraged to avoid contact with each other, the impact of this is likely limited. Finally, environmental sampling was not conducted. This could have revealed hidden reservoirs of *M. abscessus* and been useful to identify sources of infection. While we included all high-quality public *M. abscessus* data to try to help address this, corresponding metadata was limited. We speculate that the deposited sequences reflect sampling bias towards clinical rather than environmental organisms.

There is extensive diversity in global *M. abscessus* complex organisms, even though closely related isolates have been identified in patients with CF and non-CF patients at geographically distant sites^15,21,41,42^. Isolates from patients in different countries were often more closely related to each other than those from the same centre in Ontario^21^. While there is a sampling bias towards the UK in public databases, this is unlikely to affect our interpretation of the diversity of Ontario *M. abscessus* and we note that observation of UK isolates spread across all three subspecies trees does not necessarily mean that the UK is a global source of *M. abscessus* infection. To date, corresponding epidemiological data on patient contact is limited and is critical to support patient-to-patient transmission^18,39^. While our data does not demonstrate person-to-person transmission of *M. abscessus*, it is not possible to comment on the extent to which existing infection prevention and control measures impact infection acquisition. The use of negative pressure rooms and isolation is associated with high social and economic costs^45^. Further studies at other CF centres will be needed to investigate whether our results can be replicated widely and, whether infection prevention and control efforts for these patients can be refocused to relieve pressure on limited resources such as negative pressure rooms.

## Supplementary Information

Supplementary Information 1.

Supplementary Information 2.

Supplementary Information 3.

Supplementary Information 4.

Supplementary Information 5.

Supplementary Information 6.

Supplementary Information 7.

## Data Availability

Sequencing data for this study are available under NCBI Bioproject accession PRJNA778024.
