# Supplementary material for: Genomic epidemiology of Mycobacterium abscessus in a Canadian cystic fibrosis centre
Source: Sci Rep. 2022 Sep 27;12:16116. doi: 10.1038/s41598-022-19666-8 (PMC9514693; doi:10.1038/s41598-022-19666-8)
Supplement: Supplementary file 7 — Supplementary Information 7. [file 41598_2022_19666_MOESM7_ESM.docx]

**Genomic Epidemiology of *Mycobacterium abscessus* in a Canadian Cystic Fibrosis Centre**

**Authors and Affiliations**

Nicholas Waglechner^1^, Elizabeth Tullis^2,3^, Anne L. Stephenson^2,3,4^, Valerie Waters^5^, Fiona McIntosh^6,7^, Jennifer Ma^8^, Frances B. Jamieson^8^, Marcel A. Behr^6,7,9^, Jane Batt*^10,11^, Robyn S. Lee*^1,12^

^1^Dalla Lana School of Public Health, University of Toronto, 155 College St., Toronto, Ontario, Canada M5T 3M7. ^2^Adult Cystic Fibrosis Program, Division of Respirology, St. Michael’s Hospital, Unity Health Toronto, Toronto, Ontario, Canada. ^3^Li Ka Shing Knowledge Institute, St. Michael’s Hospital, Toronto, Ontario, Canada.

^4^Institute of Health Policy, Management and Evaluation, University of Toronto, Toronto, Ontario, Canada. ^5^Hospital for Sick Children, Department of Pediatrics, Division of Infectious Diseases, 555 University Avenue, Toronto, Ontario, Canada M5G 1X8. ^6^Infectious Diseases and Immunity in Global Health Program, Research Institute of the McGill University Health Centre, Montreal, Quebec, Canada. ^7^McGill International TB Centre, McGill University, Montreal, Quebec, Canada. ^8^Public Health Ontario, Public Health Ontario Laboratories, 661 University Avenue, Suite 1701, Toronto, ON, Canada M5G 1V2. ^9^Department of Epidemiology, Biostatistics and Occupational Health, McGill University, Montreal, Quebec. ^10^Keenan Research Center for Biomedical Science, St. Michael’s Hospital, Toronto, Ontario, Canada. ^11^Tuberculosis Program, St. Michael’s Hospital Unity Health Toronto, Toronto, Ontario, Canada M5B 1WB. ^12^Center for Communicable Disease Dynamics, Harvard School of Public Health, Boston, MA, USA.

*These authors contributed equally

**Supplementary Material**

Supplementary Tables

**Supplementary Table 1: Isolate Sequencing statistics and quality control.**

See attached file.

**Supplementary Table 2: Ontario isolate SNV statistics**

See attached file.

**Supplementary Table 3: Public SRA sequences used for global comparison.**

See attached file.

**Supplementary Table 4: Possible transmission pairs**

See attached file.

**Supplementary Table 5: SRA sample statistics and quality control.**

See attached file.

**Supplementary Table 6: Global *M. abscessus* isolate SNV statistics.**

See attached file

# Supplementary Figure Legends

**Supplementary Figure 1 - Assembly contiguity vs length.** Four isolates are outliers with respect to both length and number of contigs (orange) compared to the other isolates (blue).

**Supplementary Figure 2 – Pairwise distance matrix of *M. abscessus* Isolates.** Number of SNVs between each pair of isolates from the recombination masked SNV alignment used to generate the maximum-likelihood phylogeny in Figure 2.

**Supplementary Figure 3 – Pairwise distance matrix of *M. massiliense* isolates.** Number of SNVs between each pair of isolates from the recombination masked SNV alignment used to generate the maximum-likelihood phylogeny in Figure 2.

**Supplementary Figure 4 – Timeline of interactions between patients.** **a** Patients 1 and 4. **b** Patients 1 and 22. **c** Patients 4 and 22. For each patient, available visit data, microbiology results, and isolate data are indicated. Hospital visits are shown on the timeline as bars for clinic (gold), pulmonary function testing (PFT, purple), hospitalization (blue) events. Spirometry during CF clinic visits occurs in the patient clinic room, post-2014, rather than a central location. Contact recorded as a grey vertical bar for overlapping visits when they occur on the same day for both patients. *Mycobacterium-*specific microbiology results are indicated by circles for no growth (open grey), growth of other *Mycobacterium* spp. (filled grey) and growth of *Mycobacterium abscessus* complex organisms (filled green). Sequencing of collected isolates is indicated for unsequenced (open dark red) and sequenced (filled red) isolates and labelled with the isolate identifier.

**Supplementary Figure 5 – Maximum-likelihood unadjusted SNV phylogeny of global *M. abscessus* isolates.** This phylogeny includes a total 2669 *M. abscessus* isolates. The country of sampling, where available is indicated by the colour used for leaf branches and on the outer ring. The positions of Ontario patients are indicated.

**Supplementary Figure 6 – Maximum-likelihood unadjusted SNV phylogeny of global *M. massiliense* isolates.** This phylogeny includes a total 983 *M. massiliense* isolates. The country of sampling, where available is indicated by the colour used for leaf branches and on the outer ring. The positions of Ontario patients are indicated. Country colours follow Supplementary Figure 5.

**Supplementary Figure 7 – Maximum-likelihood unadjusted SNV phylogeny of *M. bolletii* isolates.** This phylogeny includes a total 219 *M. bolletii* isolates. The country of sampling, where available, is indicated by the colour used for leaf branches and on the outer ring. The position of the Ontario patient is indicated. Country colours follow Supplementary Figure 5.

# Supplementary Figures

**Supplementary Figure 1**


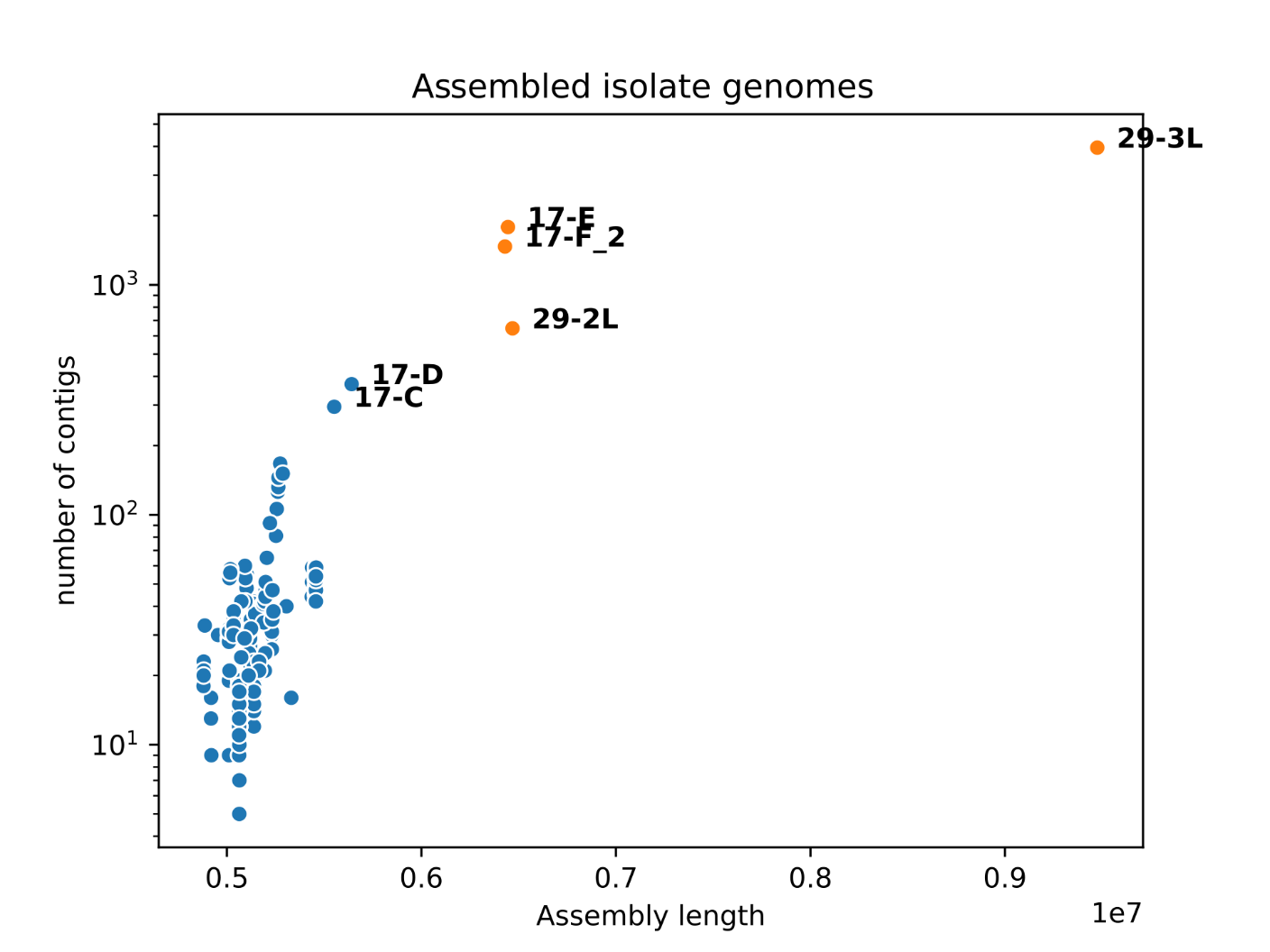


**Supplementary Figure 2**


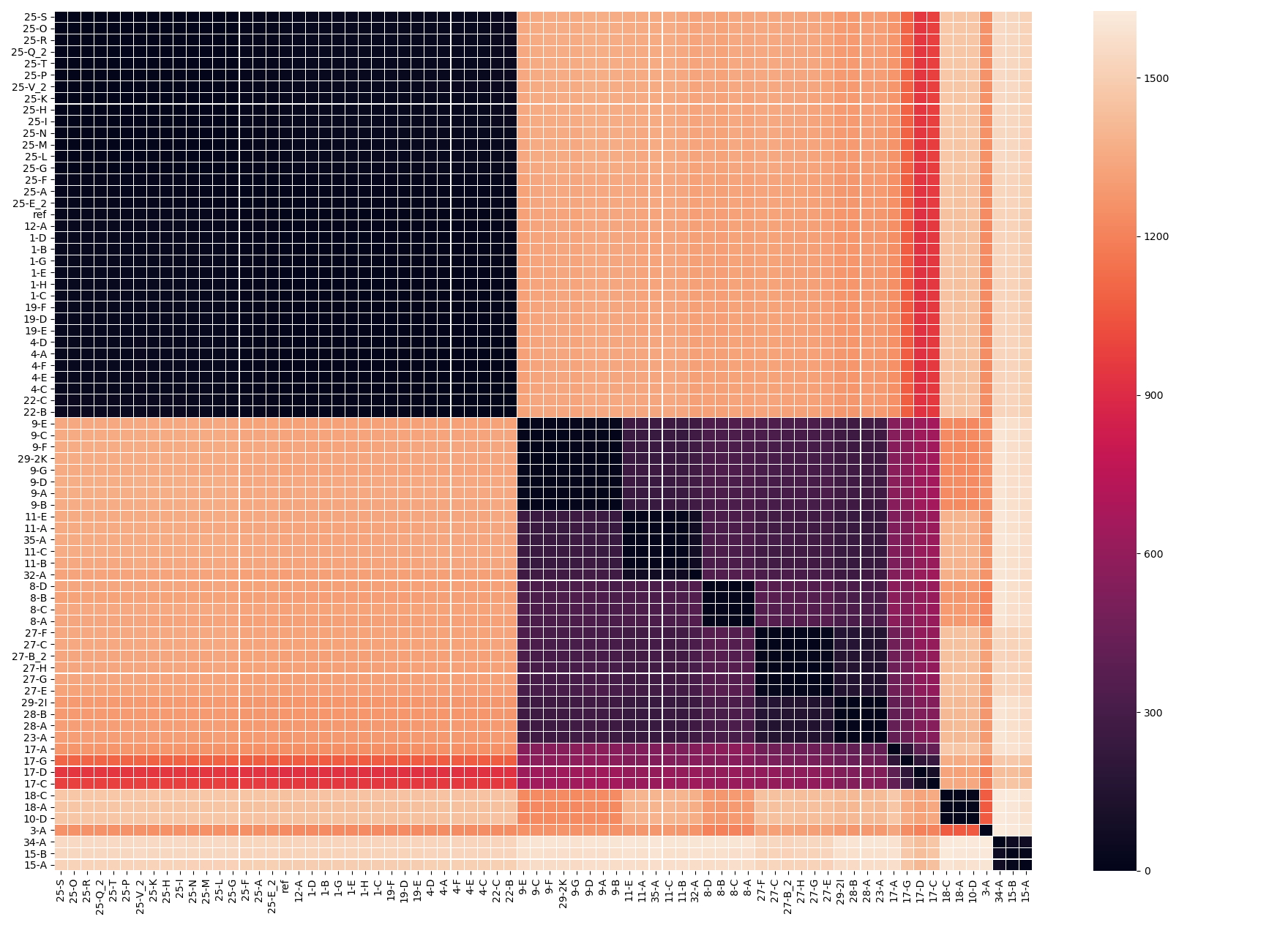


**Supplementary Figure 3**

**
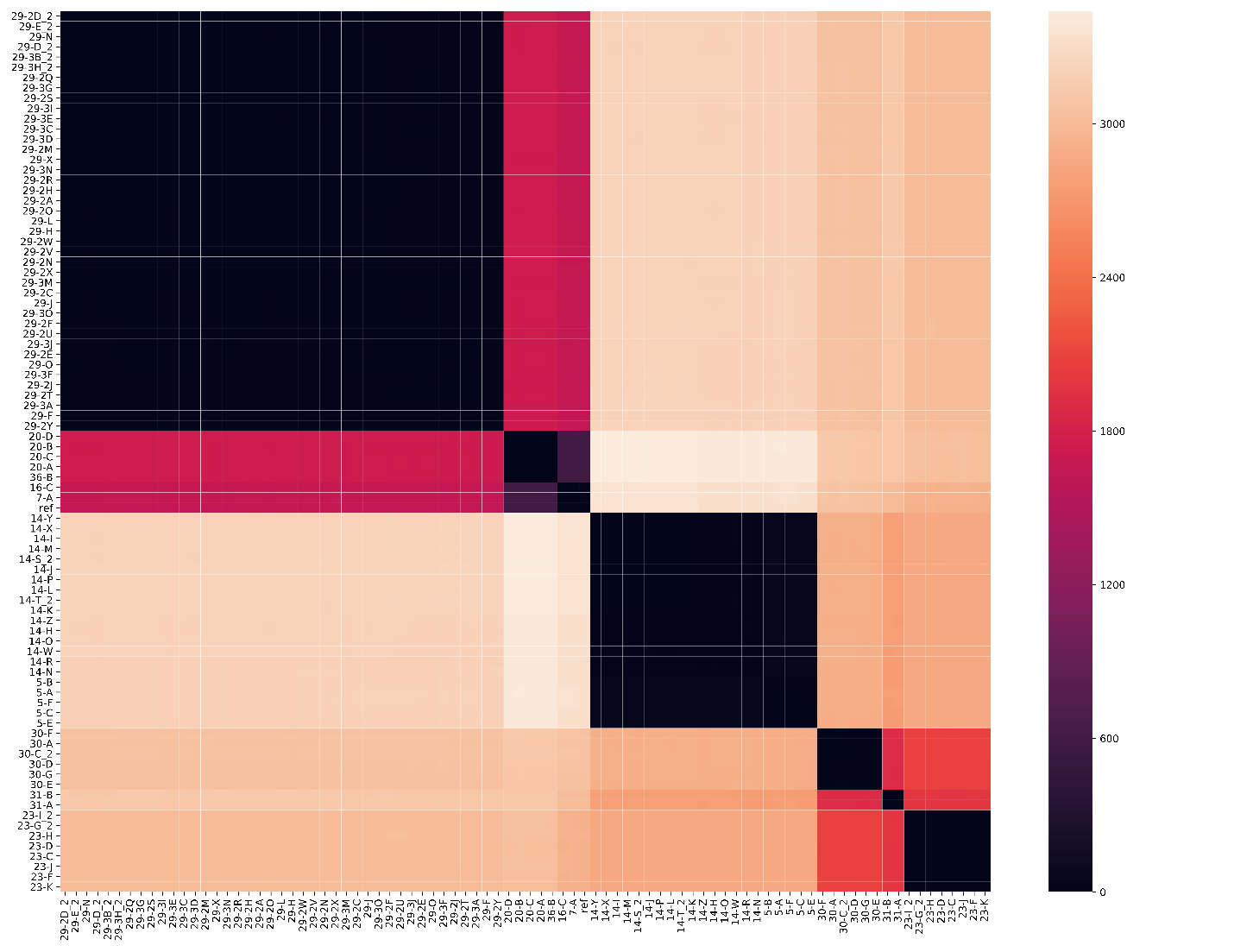
**

**Supplementary Figure 4**

**
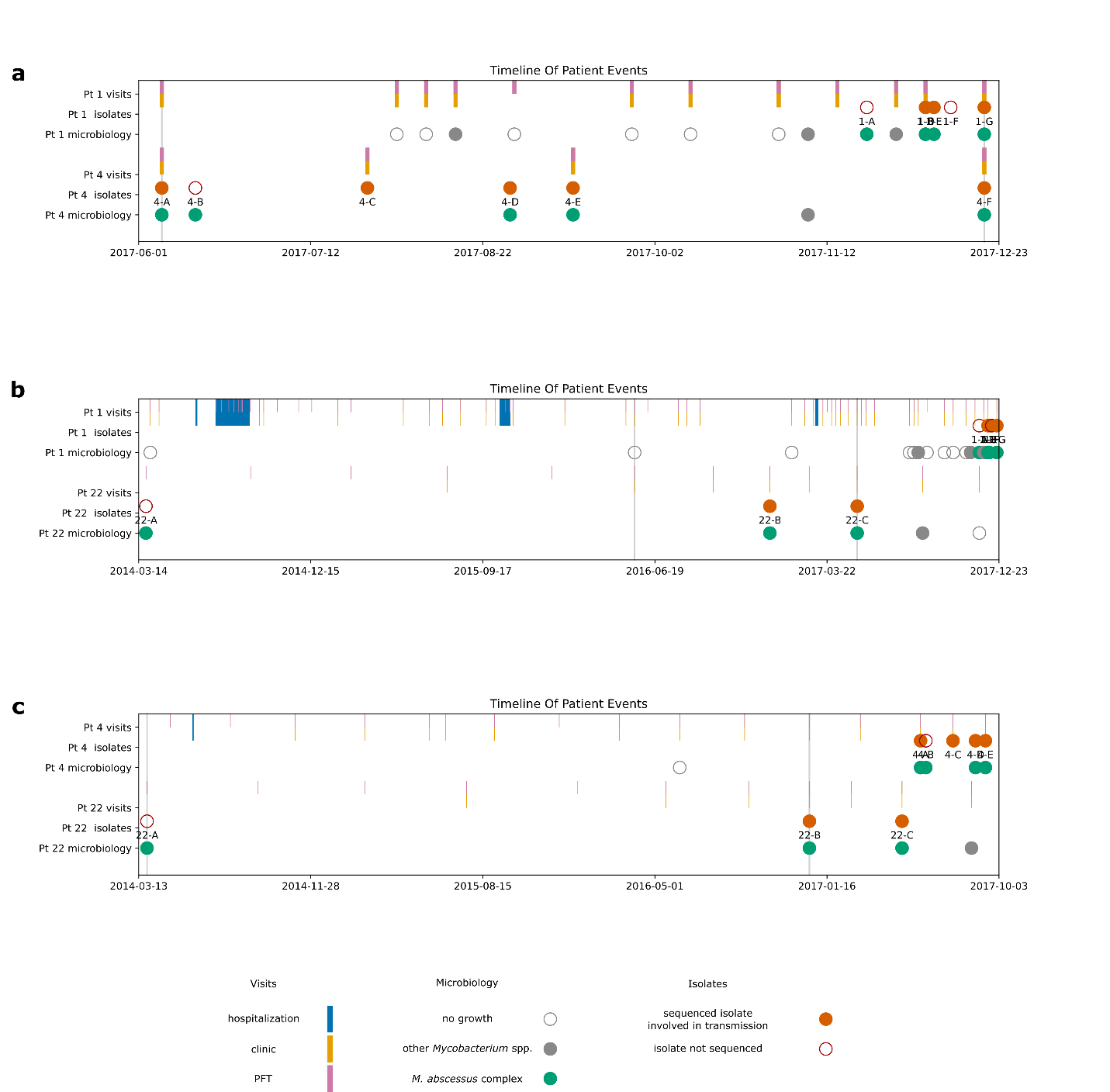
**

**Supplementary Figure 5**

**
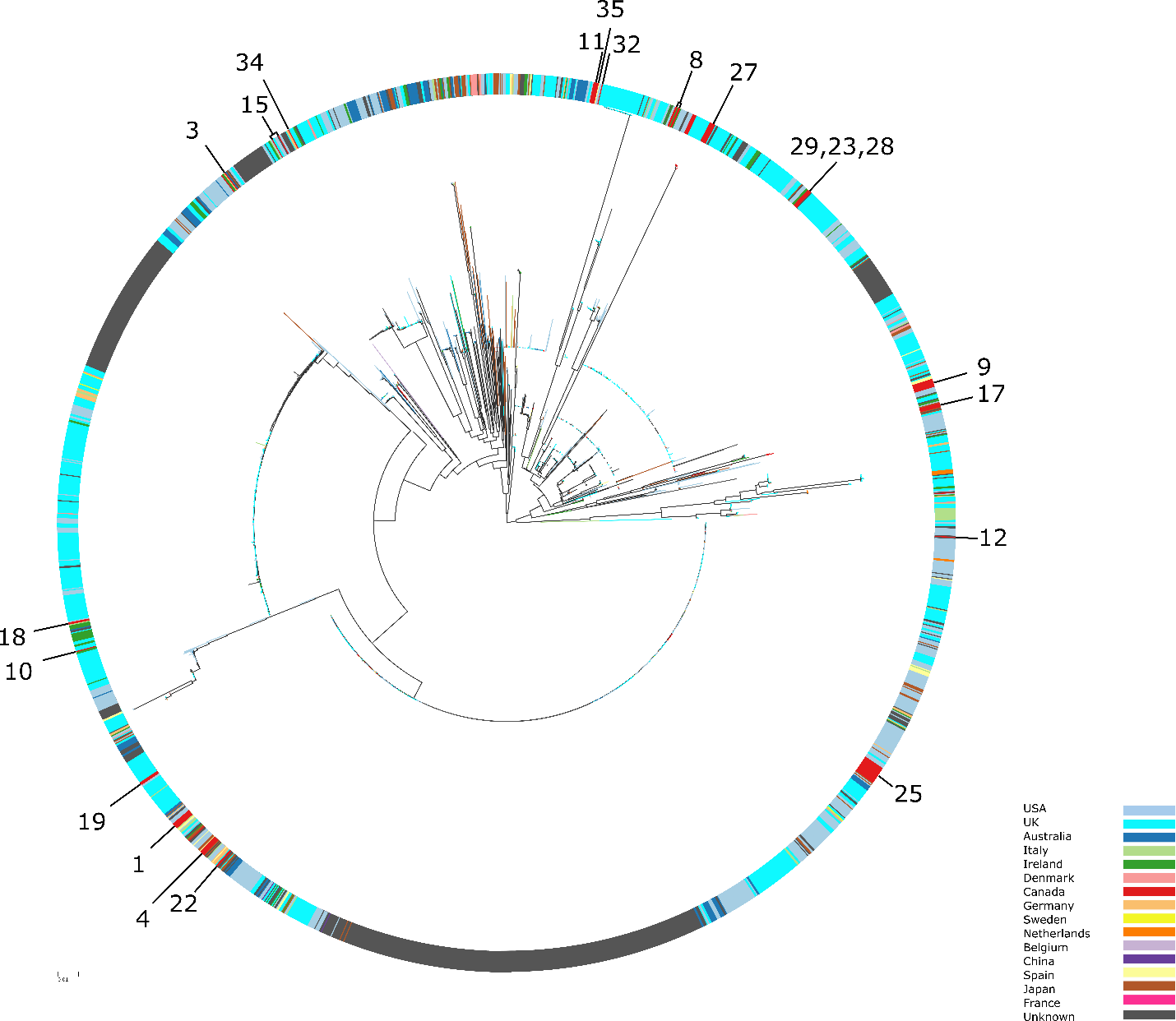
**

**Supplementary Figure 6**

**
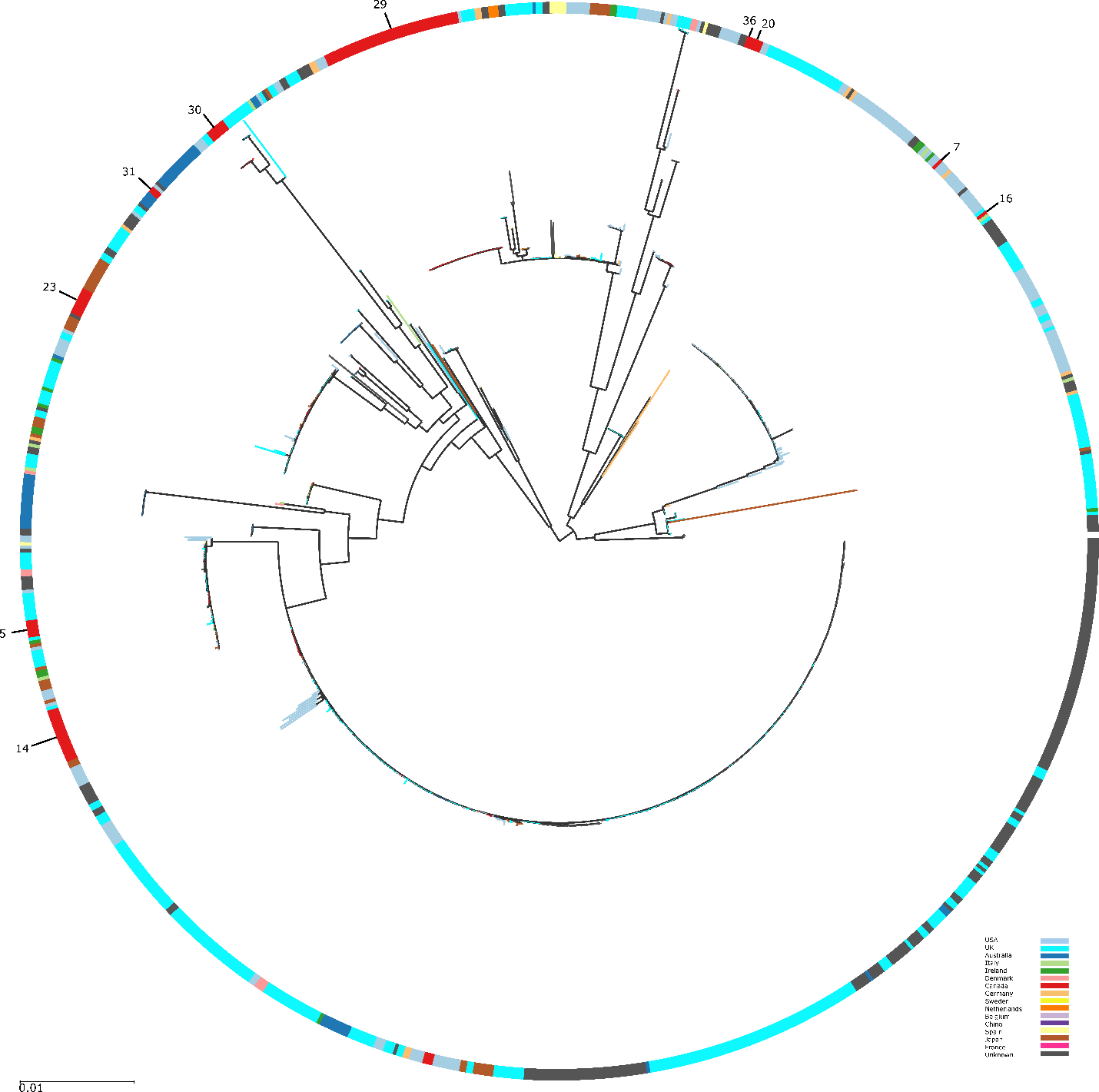
**

**Supplementary Figure 7**

**
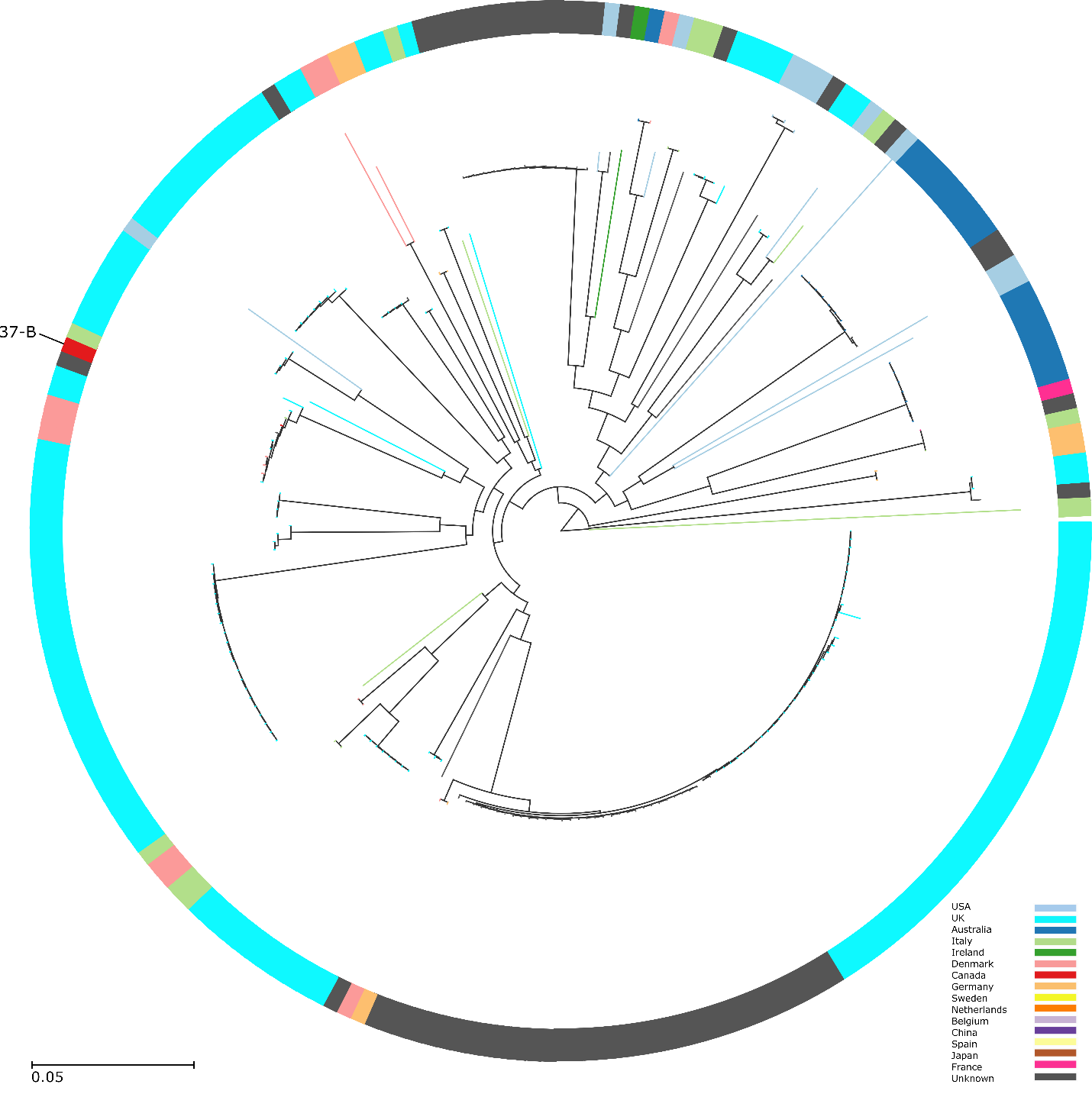
**

**Supplementary Figure 8**

**
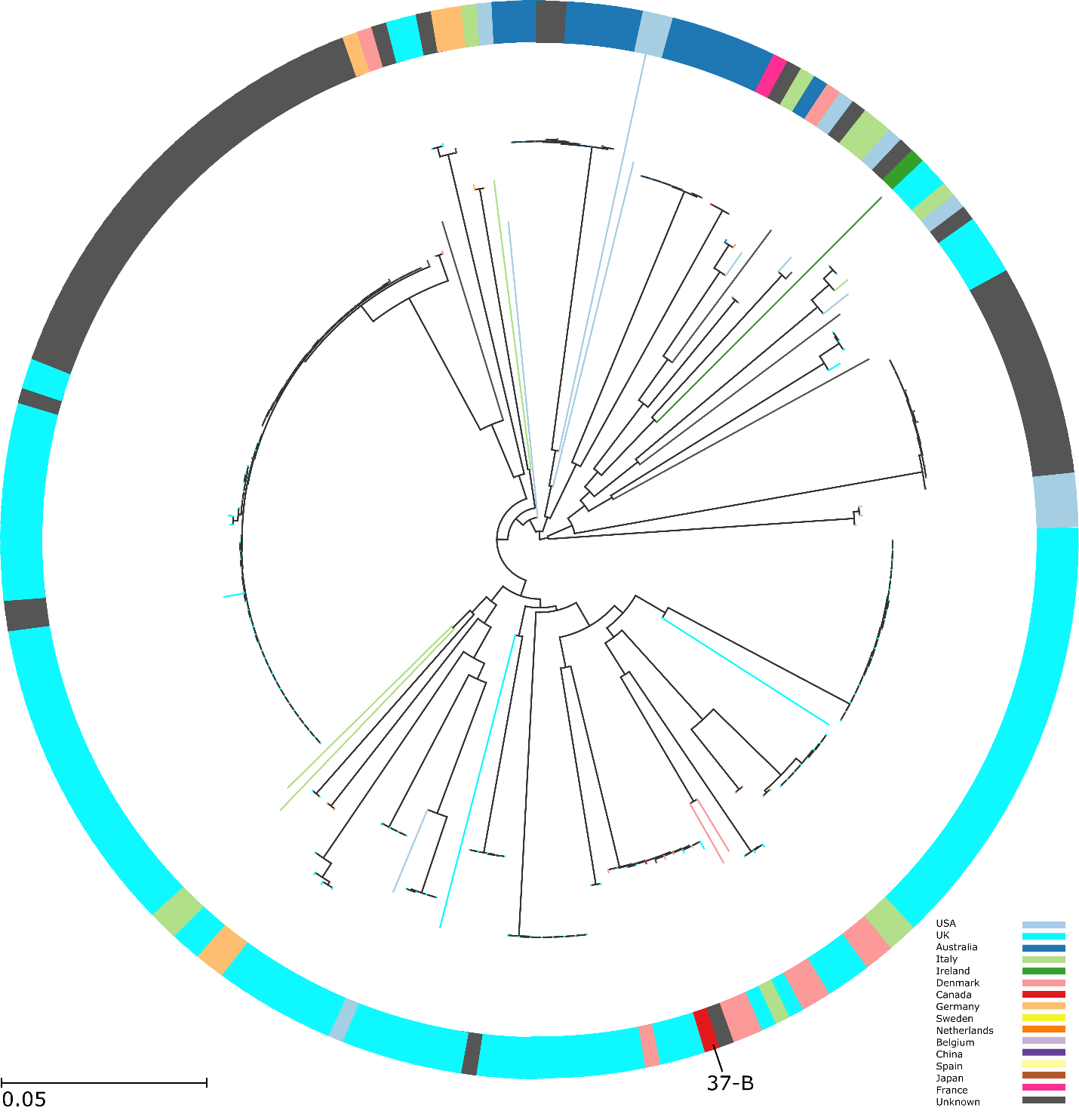
**
